# Supplementary material for: A single-cell transcriptional timelapse of mouse embryonic development, from gastrula to pup
Source: bioRxiv. 2023 Apr 5:2023.04.05.535726. Preprint. [Version 1] doi: 10.1101/2023.04.05.535726 (PMC10104014; doi:10.1101/2023.04.05.535726)
Supplement: Supplement 2 [file NIHPP2023.04.05.535726v1-supplement-2.pdf]

bioRxiv preprint doi: https://doi.org/10.1101/2023.04.05.535726; this version posted April 5, 2023. The copyright holder for this preprint (which was not certified by peer review) is the author/funder, who has granted bioRxiv a license to display the preprint in perpetuity. It is made available under aCC-BY 4.0 International license.

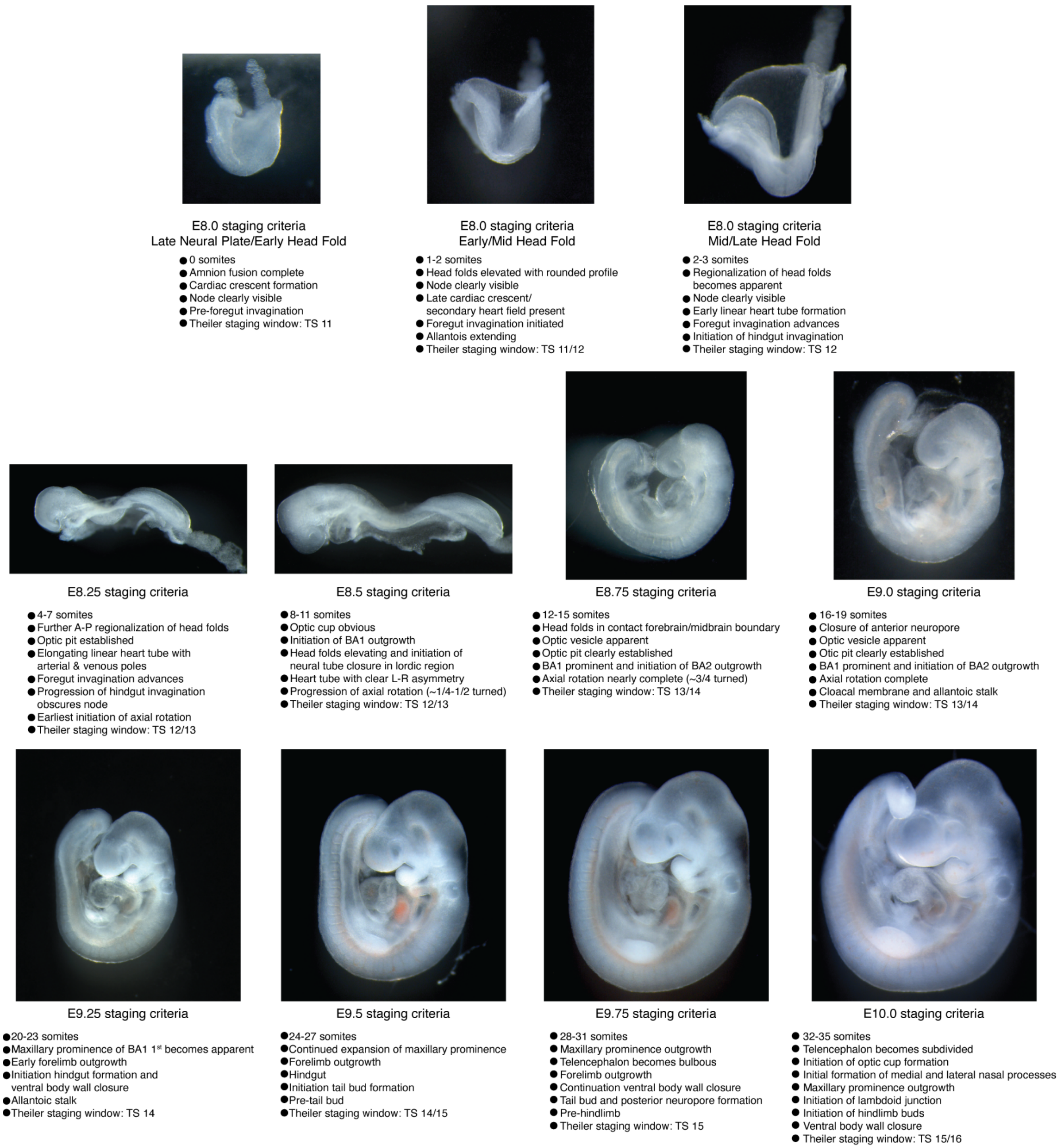

Supplementary Figure 1. Embryos harvested between E8 and E10 were precisely staged based upon somite counting. Harvested embryos were grouped into bins based on somite counting and further characterized based upon morphological features. Stage-representative images are shown with details of the main staging criteria for each coarse temporal bin listed. The approximately overlapping Theiler Stage (TS) is also noted for reference.

bioRxiv preprint doi: https://doi.org/10.1101/2023.04.05.535726; this version posted April 5, 2023. The copyright holder for this preprint (which was not certified by peer review) is the author/funder, who has granted bioRxiv a license to display the preprint in perpetuity. It is made available under aCC-BY 4.0 International license.

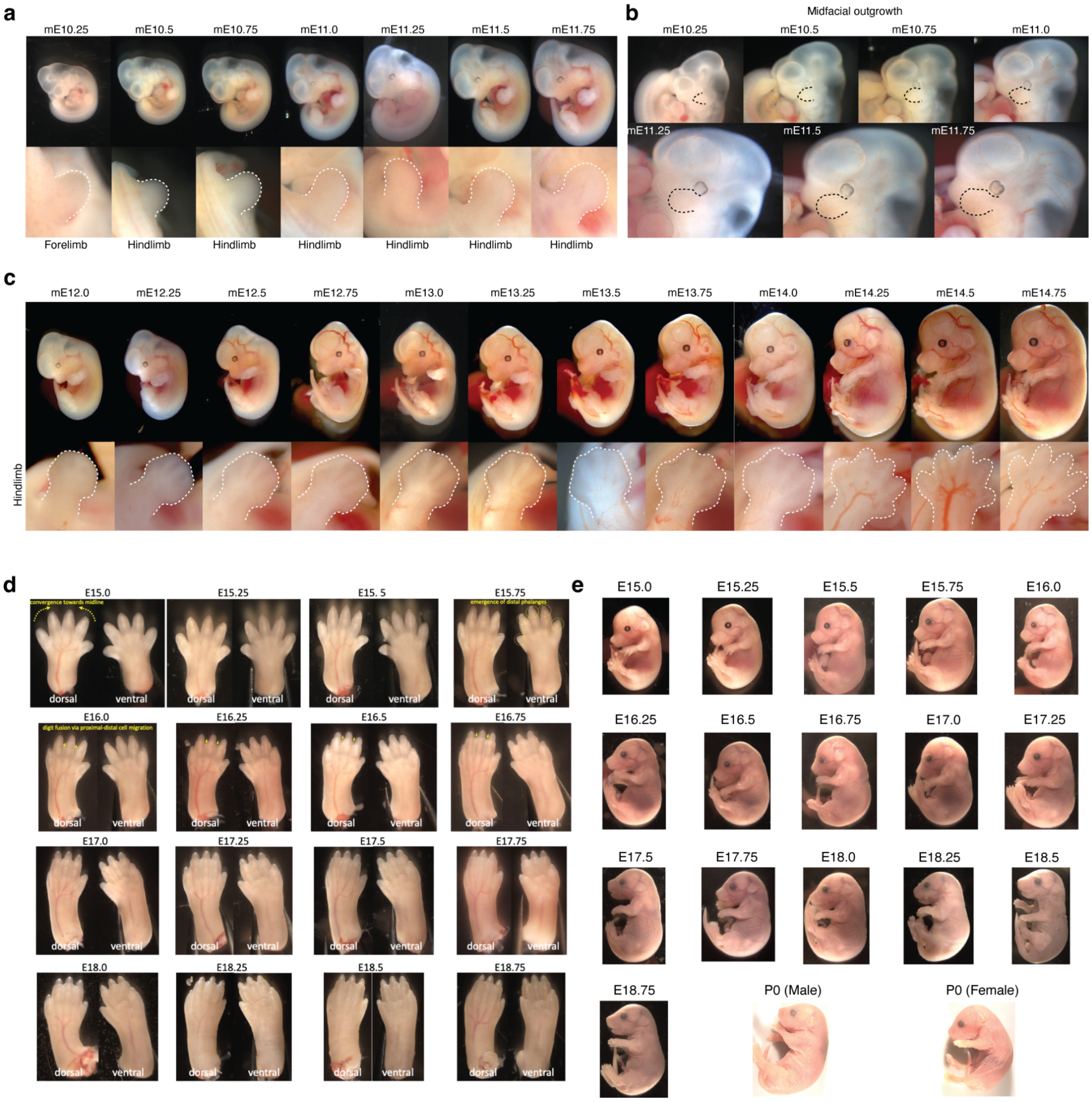

Supplementary Figure 2. After E10, embryos were precisely staged based on morphological features. This was mainly done using the embryonic mouse ontogenetic staging system (eMOSS), an automated process that leverages limb bud geometry to infer developmental stage[15,16]. Staging results derived from eMOSS are designated with "mE" for morphometric embryonic day. **a,** For each temporal bin at 6-hr increments from E10.25-E11.75, an image of a stage-representative embryo is shown in the top row. Images of each embryo's limb bud (white dashed outline) used for staging are shown in the bottom row. **b,** View of the craniofacial region of embryos shown in panel **a** demonstrates that limb bud staging also recreates the ordered ontogenetic progression of craniofacial morphogenesis, including development of the brain, eye, and outgrowth of facial prominences (black dashed line highlights maxillary process). **c,** For each temporal bin at 6-hr increments from E12.0-E14.25, an image of a randomly selected embryo is shown in the top row. The subview of its hindlimb is shown in the bottom row. **d,** eMOSS is able to stage E10.25-E4.75, after which limb morphology becomes too complex. We defined additional dynamics related to digit formation to stage E15.0-E16.75 embryos. However, the remaining timepoints (E17.0-E18.75) were staged based upon gestational age. For each temporal bin at 6-hr increments from E15.0-E18.75, an image of the hindlimbs of a randomly selected embryo is shown. **e,** For each temporal bin at 6-hr increments from E15.0-P0, an image of a stage-representative embryo is shown.

bioRxiv preprint doi: https://doi.org/10.1101/2023.04.05.535726; this version posted April 5, 2023. The copyright holder for this preprint (which was not certified by peer review) is the author/funder, who has granted bioRxiv a license to display the preprint in perpetuity. It is made available under aCC-BY 4.0 International license.

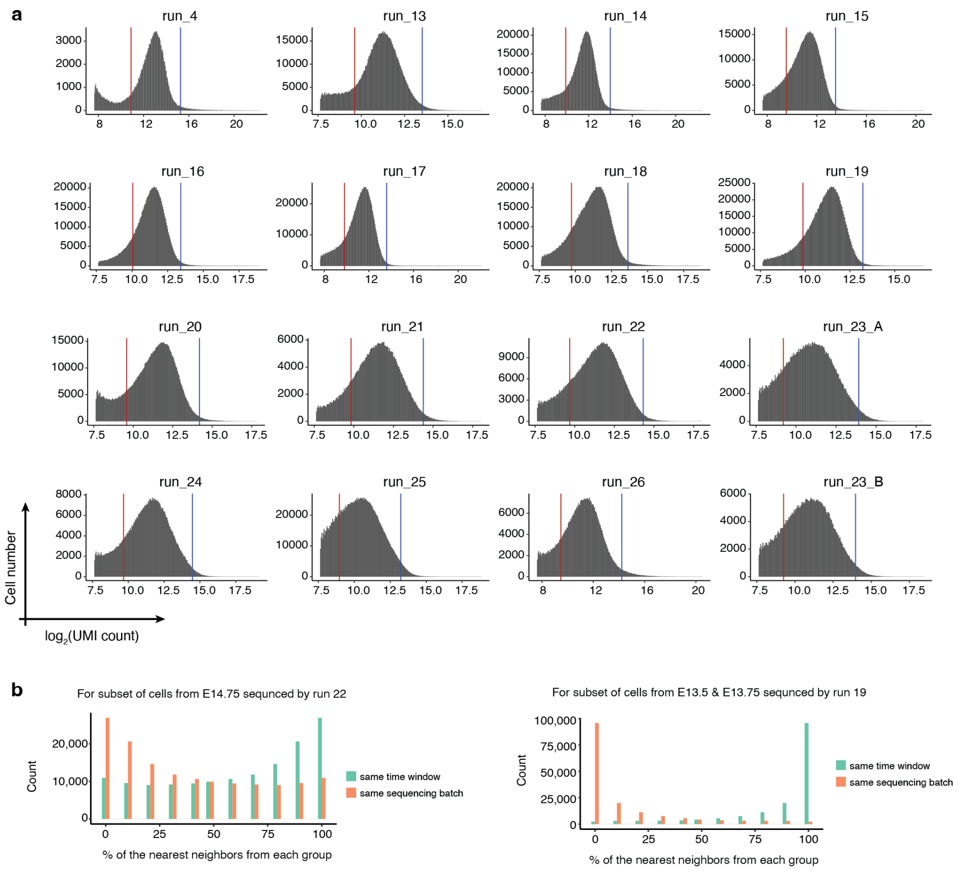

Supplementary Figure 3. Higher quality sci-RNA-seq3 data as generated by an optimized protocol. a, Histograms of $\log_2$(UMI count) per single nucleus for each of 15 sci-RNA-seq3 experiments. For the 14 newly performed experiments (run_13 to run_26), upper (blue line) and lower (red line) thresholds used for quality filtering correspond to the mean plus 2 standard deviations and mean minus 1 standard deviation of log2-scaled values, respectively, after excluding cells with >85% of reads mapping to exonic regions (except for the lower bound of 500, which was manually assigned for run_25), are shown with vertical lines. The data of run_4, which was reported previously[11], was subjected to the same thresholds used in the original study, *i.e.* the mean +/- 2 standard deviations of log2-scaled values (blue and red vertical lines, respectively), after excluding cells with >85% of reads mapping to exonic regions. Run_23_A & B were from the same sci-RNA-seq3 experiment, but with nuclei which were sequenced separately. **b,** Although most of the embryos from the same approximate stage (*e.g.* E14.0-E14.75) were included in the same sci-RNA-seq3 experiment (**Supplementary Table 1**), we profiled extra nuclei in some experiments for a handful of timepoints to ensure sufficient coverage. Here we sought to leverage those instances to check for potential batch effects across experiments. For this, on the embedding learned from all of the data, we asked whether these cells' profiles are more similar to cells from the same experiment or, alternatively, cells from the same time window. Left: for a random subset of cells from E14.75 which were profiled in experiment run_22 (primarily E17.0-E17.75), we performed a *k*-nearest neighbors (*k*NN, k = 10) approach in the global 3D UMAP to find the nearest neighboring cells either from the same experiment (red) or the same time window (E14.0-E14.75) but different experiment (blue). The percentages of the nearest neighboring cells from the two groups for individual cells are presented in the histogram. Right: for a random subset of cells from E13.5 & E13.75 which were profiled in experiment run_19 (primarily E10.5-E11), we performed a *k*-nearest neighbors (*k*NN, k = 10) approach in the global 3D UMAP to find the nearest neighboring cells either from the same experiment (red) or the same time window (E13.0-E13.75) but a different experiment (blue). The percentages of the nearest neighboring cells from the two groups for individual cells are presented in the histogram. In both examples, we observe that nearest neighbors are overwhelmingly cells from a different experiment (but the same time window), rather than cells from the same experiment (but a different time window).

bioRxiv preprint doi: https://doi.org/10.1101/2023.04.05.535726; this version posted April 5, 2023. The copyright holder for this preprint (which was not certified by peer review) is the author/funder, who has granted bioRxiv a license to display the preprint in perpetuity. It is made available under aCC-BY 4.0 International license.

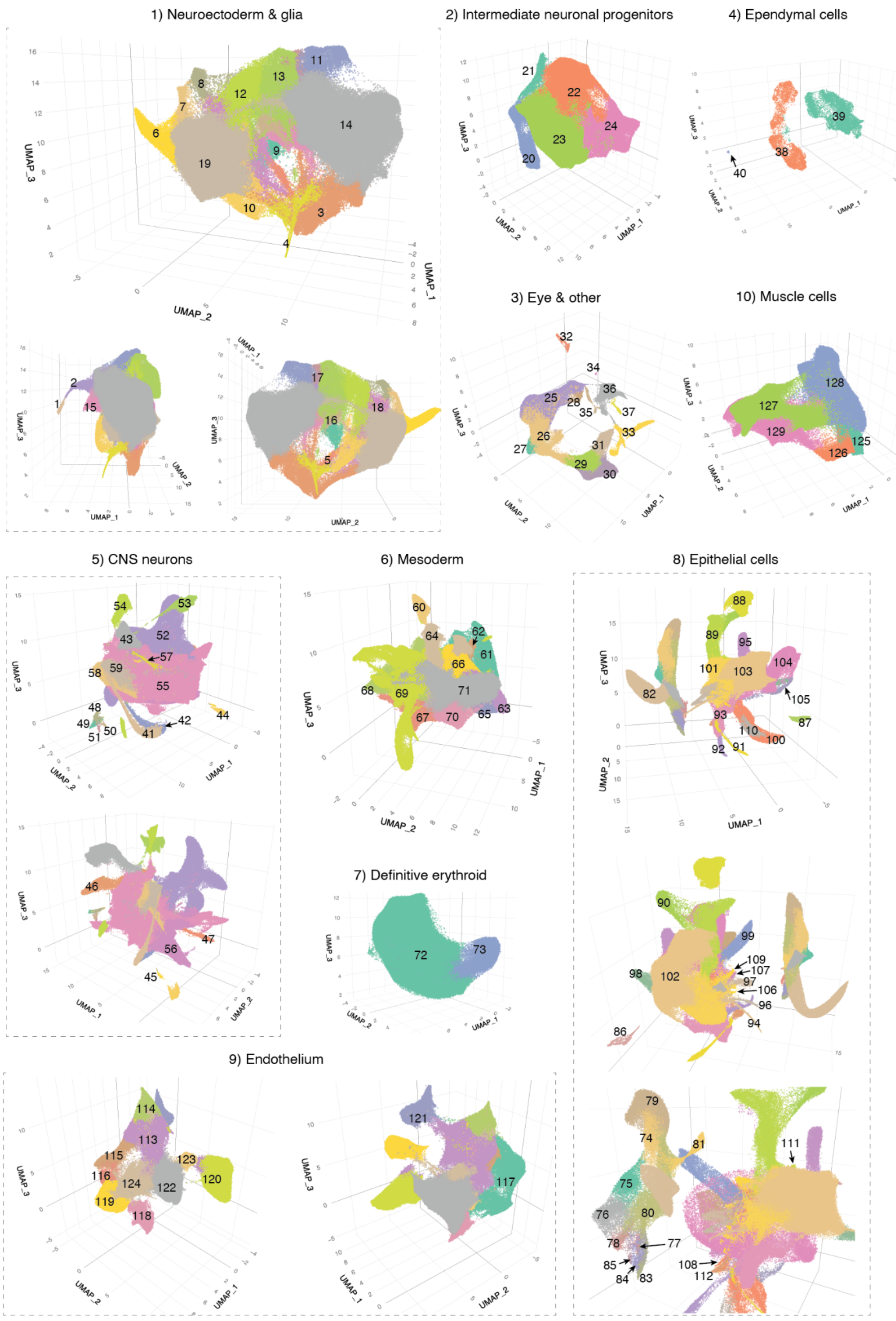

1 - Retinal pigment cells
2 - Eye field
3 - Astrocytes
4 - Multiciliated ependymal cells
5 - Cerebellum-related cells
6 - NMPs & spinal cord progenitors
7 - Posterior roof plate
8 - Anterior roof plate
9 - Anterior floor plate
10 - Floorplate and p3 domain
11 - Dorsal telencephalon
12 - Midbrain-hindbrain boundary
13 - Midbrain
14 - Telencephalon
15 - Hypothalamus
16 - Hypothalamus (Sim1+)
17 - Diencephalon
18 - Hindbrain
19 - Spinal cord/r7/r8
20 - Intermediate neuronal progenitors
21 - Cortical Interneurons (Prox1+)
22 - Deep-layer neurons
23 - Upper-layer neurons
24 - Subplate neurons
25 - Naive retinal progenitor cells
26 - Retinal progenitor cells
27 - Bipolar precursor cells
28 - Ciliary margin cells
29 - Photoreceptor precursor cells
30 - Cone precursor cells
31 - Rod precursor cells
32 - Cochlear hair cells
33 - Pituitary gland cells
34 - Nonsensory cochlear epithelium
35 - Pancreatic islets
36 - Suprachiasmatic nucleus
37 - Pineal gland
38 - Ependymal cells
39 - Choroid plexus
40 - Ciliated nodal cells
41 - Spinal cord motor neurons
42 - Cranial motor neurons
43 - Thalamic neuronal precursors
44 - Retinal ganglion cells
45 - PV-containing retinal ganglion cells
46 - Cajal-Retzius cells
47 - Cerebellar Purkinje cells
48 - Amacrine/Horizontal precursor cells
49 - Amacrine cells
50 - Horizontal cells
51 - Cholinergic amacrine cells
52 - GABAergic cortical interneurons
53 - Neural progenitor cells (Neurod1+)
54 - Neural progenitor cells (Ror1+)
55 - Glutamatergic neurons
56 - GABAergic neurons
57 - Neurons (Slc17a8+)
58 - Spinal cord dorsal progenitors
59 - Spinal cord ventral progenitors
60 - Mesodermal progenitors (Tbx6+)
61 - Chondrocytes (Atp1a2+)
62 - Chondrocytes (Otor+)
63 - Early chondrocytes
64 - Pre-osteoblasts (Sp7+)
65 - Dermomyotome
66 - Limb mesenchyme progenitors
67 - Dermatome
68 - Fibroblasts
69 - Lateral plate and intermediate mesoderm
70 - Facial mesenchyme
71 - Sclerotome
72 - Definitive early erythroblasts (CD36-)
73 - Definitive erythroblasts (CD36+)
74 - Posterior intermediate mesoderm
75 - Anterior intermediate mesoderm
76 - Ureteric bud
77 - Collecting duct intercalated cells
78 - Collecting duct principal cells
79 - Metanephric mesenchyme
80 - Nephron progenitors
81 - Podocytes
82 - Proximal tubule cells
83 - Ascending loop of Henle
84 - Distal convoluted tubule
85 - Connecting tubule
86 - Lens epithelial cells
87 - Notochord
88 - Otic epithelial cells
89 - Olfactory epithelial cells
90 - Olfactory bulb cells
91 - Pancreatic acinar cells
92 - Foregut epithelial cells
93 - Gut
94 - Primordial germ cells
95 - Amniotic ectoderm

bioRxiv preprint doi: https://doi.org/10.1101/2023.04.05.535726; this version posted April 5, 2023. The copyright holder for this preprint (which was not certified by peer review) is the author/funder, who has granted bioRxiv a license to display the preprint in perpetuity. It is made available under aCC-BY 4.0 International license.

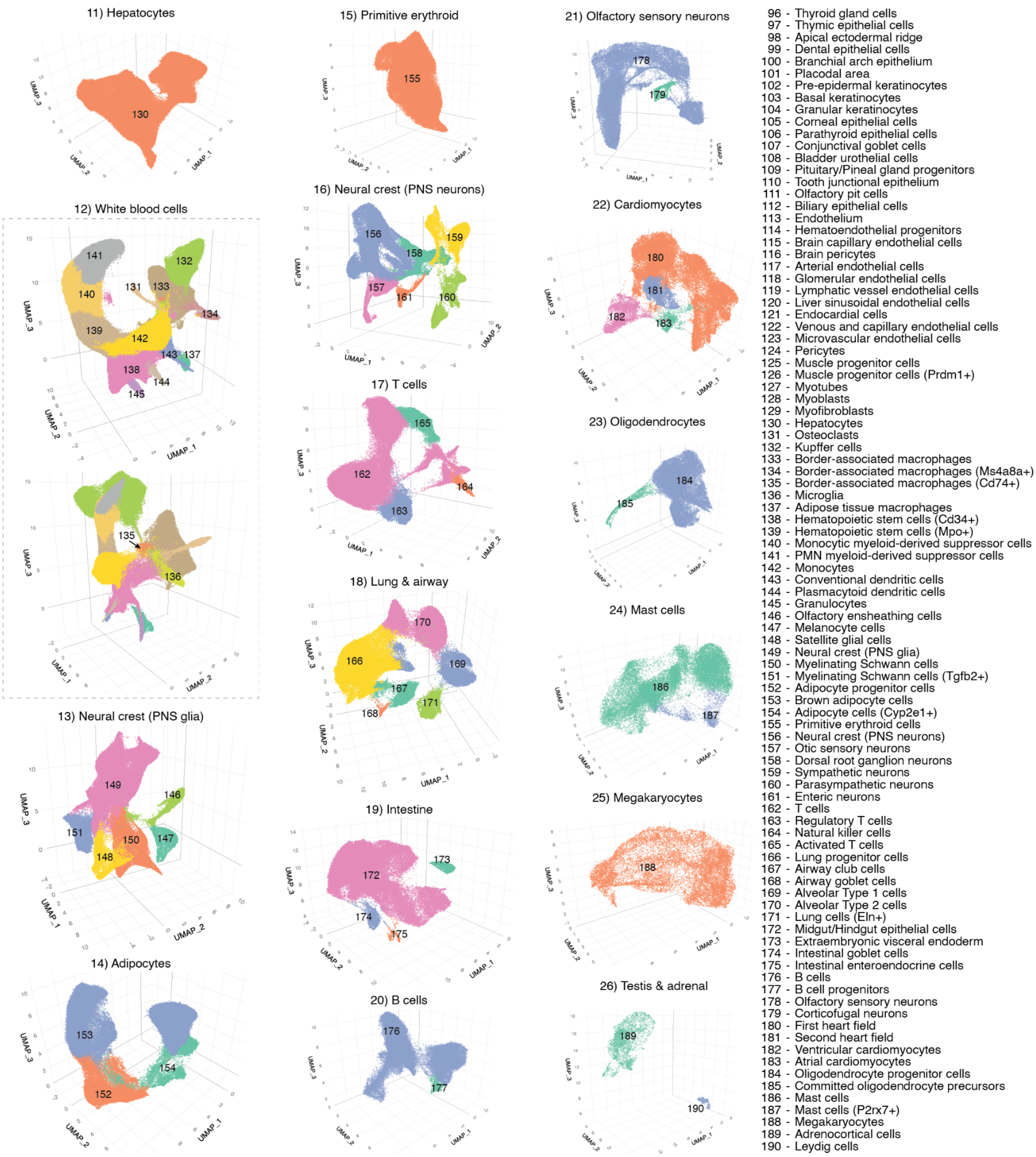

Supplementary Figure 4. Cell type annotations. For each of the 26 major cell clusters, we performed subclustering and then annotated each of 190 subclusters using at least two literature-nominated marker genes per cell type label (**Supplementary Table 5**).

bioRxiv preprint doi: https://doi.org/10.1101/2023.04.05.535726; this version posted April 5, 2023. The copyright holder for this preprint (which was not certified by peer review) is the author/funder, who has granted bioRxiv a license to display the preprint in perpetuity. It is made available under aCC-BY 4.0 International license.

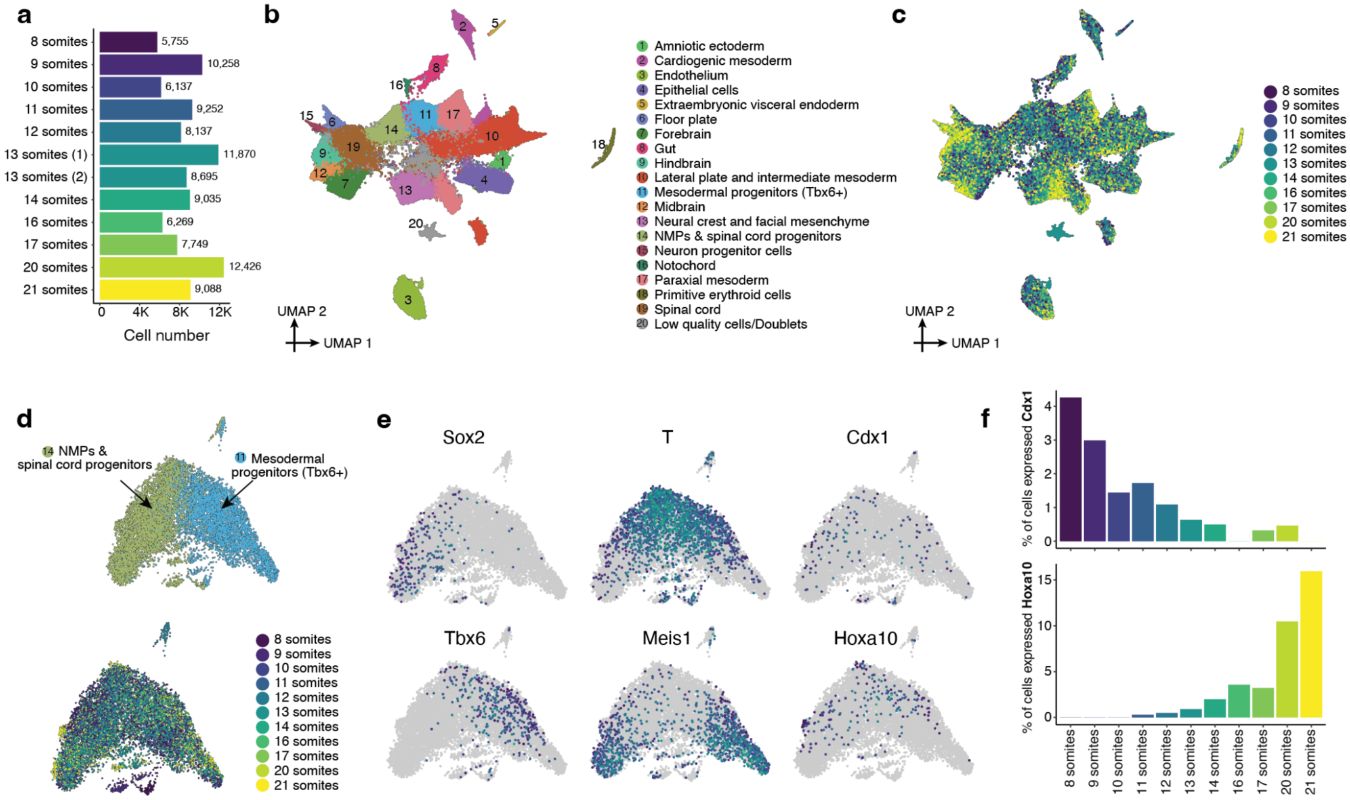

Supplementary Figure 5. A validation sci-RNA-seq3 dataset of mouse embryos from somites 8 to 21. To validate findings related to differences between embryos staged with early vs. late somite counts, particularly in NMPs, we profiled another 12 precisely staged mouse embryos, ranging from 8 to 21 somites, in an independent sci-RNA-seq3 experiment. The resulting library was sequenced on an Illumina NextSeq 2000, resulting in 104,671 cells in total, with a median UMI count of 513 and a median gene count of 446 per cell. **a,** The number of cells profiled from each embryo. **b,** 2D UMAP visualization of the validation dataset (all cell types). **c,** The same UMAP as in panel b, with cells colored by somite count of the originating embryo. **d,** Re-embedded 2D UMAP of 9,686 cells from NMPs & spinal cord progenitors (cluster 11) and mesodermal progenitors (*Tbx6*+) (cluster 14) in panel b. Cells are colored by either the original annotation (top) or somite count (bottom). **e,** The same UMAP as in panel d, colored by gene expression of marker genes which appear specific to different subpopulations of NMPs: column 1: differences between neuroectodermal (*Sox2*+) vs. mesodermal (*Tbx6*+) fates[35]; column 2: the differentiation of bipotential NMPs (*T*+, *Meis1*-) towards either fate[36,37]; column 3: earlier (*Cdx1*+) vs. later (*Hoxa10*+) NMPs[24]. **f,** Within the cells shown in panel d, the proportion of cells (y-axis) which express either *Cdx1* (top) or *Hoxa10* (bottom) are plotted as a function of somite count of the originating embryo.

bioRxiv preprint doi: https://doi.org/10.1101/2023.04.05.535726; this version posted April 5, 2023. The copyright holder for this preprint (which was not certified by peer review) is the author/funder, who has granted bioRxiv a license to display the preprint in perpetuity. It is made available under aCC-BY 4.0 International license.

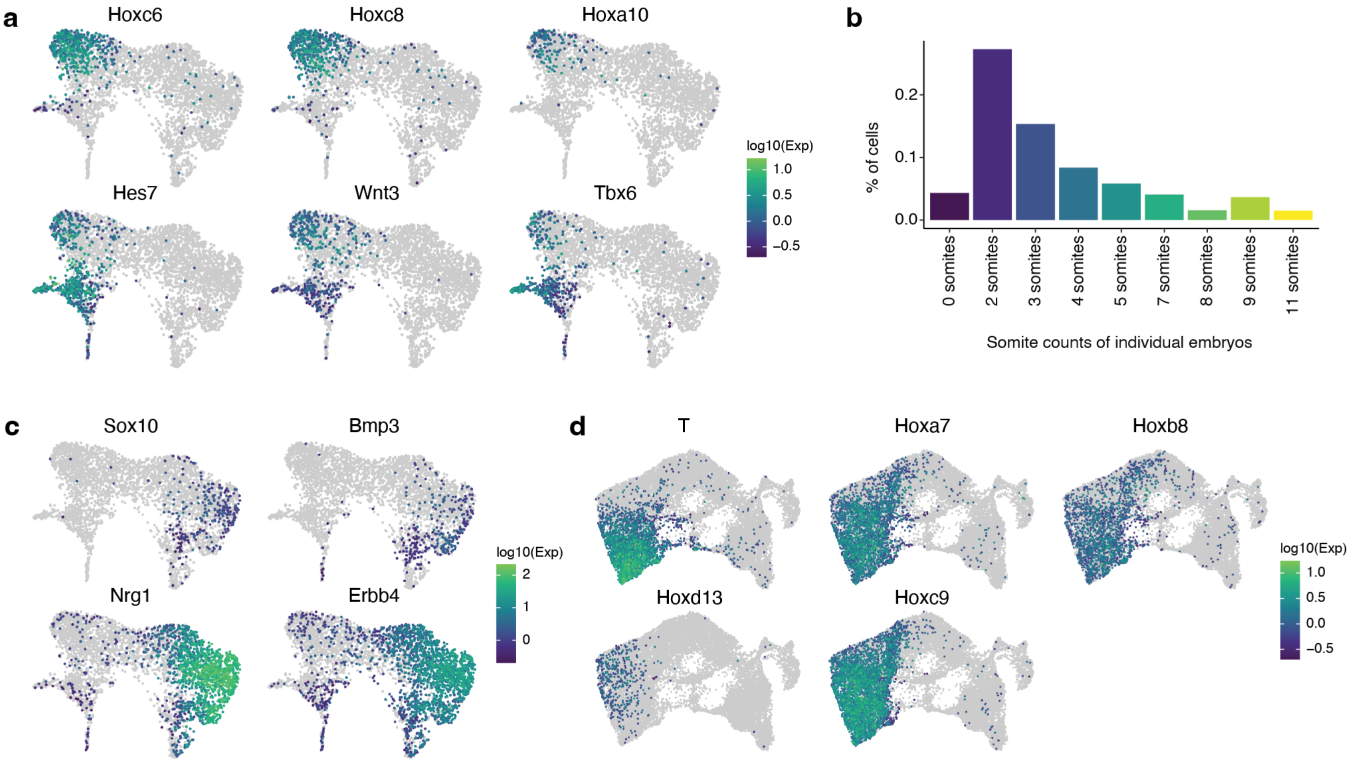

Supplementary Figure 6. Transcriptional heterogeneity in the posterior embryo during the early somitogenesis. a, The same UMAP as in **Fig. 2h**, colored by gene expression of marker genes which appear specific to the subpopulation of notochord cluster that is *Noto*+, including posterior *Hox* genes (*Hoxc6*, *Hoxc8*, *Hoxa10*), and genes involved in Notch signaling (*Hes7*), Wnt signaling (*Wnt3*) and mesodermal differentiation (*Tbx6*). **b,** Cell proportions falling into the ciliated nodal cell cluster for embryos with different somite counts. **c,** The same UMAP as in **Fig. 2h**, colored by gene expression of marker genes which appear specific to the subpopulation of the notochord *Noto*- and more strongly *Shh*+, including *Sox10*, *Bmp3*, *Nrg1*, and *Erbb4*. **d,** The same UMAP as in **Fig. 2j**, colored by gene expression of marker genes which appear specific to the posterior gut endoderm, including *T*, *Hoxa7*, *Hoxb8*, *Hoxd13*, and *Hoxc9*.

bioRxiv preprint doi: https://doi.org/10.1101/2023.04.05.535726; this version posted April 5, 2023. The copyright holder for this preprint (which was not certified by peer review) is the author/funder, who has granted bioRxiv a license to display the preprint in perpetuity. It is made available under aCC-BY 4.0 International license.

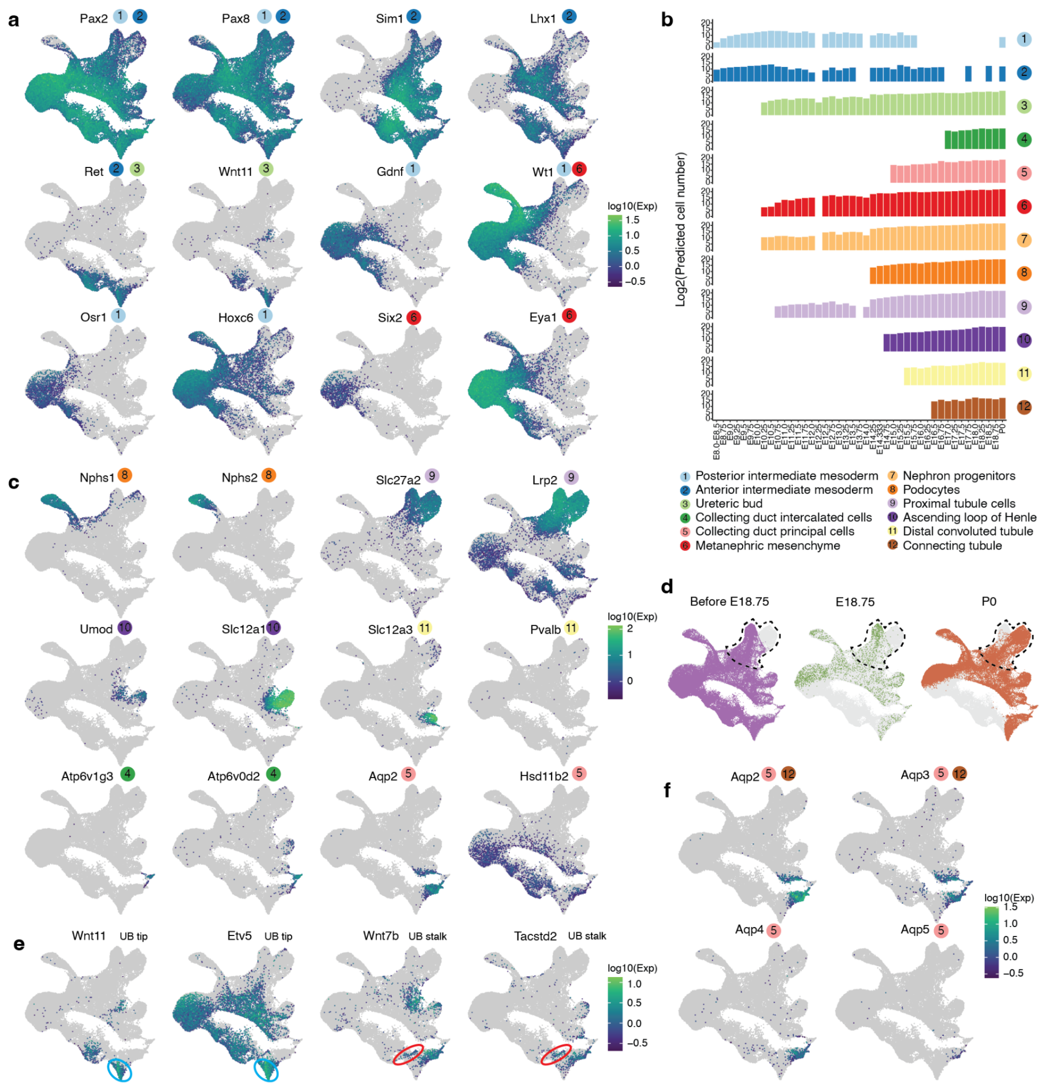

Supplementary Figure 7. Transcriptional heterogeneity in renal development. a, The same UMAP as in **Fig. 3a**, colored by expression of marker genes which appear specific to anterior intermediate mesoderm (*Pax2*+, *Pax8*+, *Sim1*+, *Lhx1*+, *Ret*+), posterior intermediate mesoderm (*Pax2*+, *Pax8*+, *Gdnf1*+, *Wt1*+, *Osr1*+, *Hoxc6*+), ureteric bud (*Ret*+, *Wnt11*+) or metanephric mesenchyme (*Wt1*+, *Six2*+, *Eya1*+). References for marker genes are provided in **Supplementary Table 5**. **b,** The predicted absolute number (log2 scale) of cells of each renal cell type at each timepoint. The predicted absolute number was calculated by the product of its sampling fraction in the overall embryo and the predicted total number of cells in the whole embryo at the corresponding timepoint (**Fig. 1e**). For each row, the first timepoint with at least 10 cells assigned that cell type annotation is labeled, and all observations prior to that timepoint are discarded. **c,** The same UMAP as in **Fig. 3a**, colored by expression of marker genes which appear specific to podocytes (*Nphs1*+, *Nphs2*+), proximal tubule cells (*Slc27a2*+, *Lrp2*+), ascending loop of Henle (*Umod*+, *Slc12a1*+), distal convoluted tubule (*Slc12a3*+, *Pvalb*+), collecting duct intercalated cells (*Atp6v1g3*+, *Atp6v0d2*+) or collecting duct principal cells (*Aqp2*+, *Hsd11b2*+). References for marker genes are provided in **Supplementary Table 5**. **d,** The same UMAP as **Fig. 3a** is shown three times, with colors highlighting cells from before E18.75 (left), E18.75 (middle), or P0 (right). Dotted cycles highlight cells which appear to correspond to the proximal tubule. **e,** The same UMAP as in **Fig. 3a**, colored by expression of marker genes which appear specific to the ureteric bud tip (*Wnt11*+, *Ret*+, *Etv5*+) or stalk (*Wnt7b*+, *Tacstd2*+)[54]. Ureteric bud tip and stalk are highlighted by blue and red circles, respectively. **f,** The same UMAP as in **Fig. 3a**, colored by expression of marker genes which appear specific to connecting tubule cells (*Aqp2*+, *Aqp3*+, *Aqp4*-) or collecting duct cells (*Aqp2*+, *Aqp3*+, *Aqp4*+, *Aqp5*+)[64].

bioRxiv preprint doi: https://doi.org/10.1101/2023.04.05.535726; this version posted April 5, 2023. The copyright holder for this preprint (which was not certified by peer review) is the author/funder, who has granted bioRxiv a license to display the preprint in perpetuity. It is made available under aCC-BY 4.0 International license.

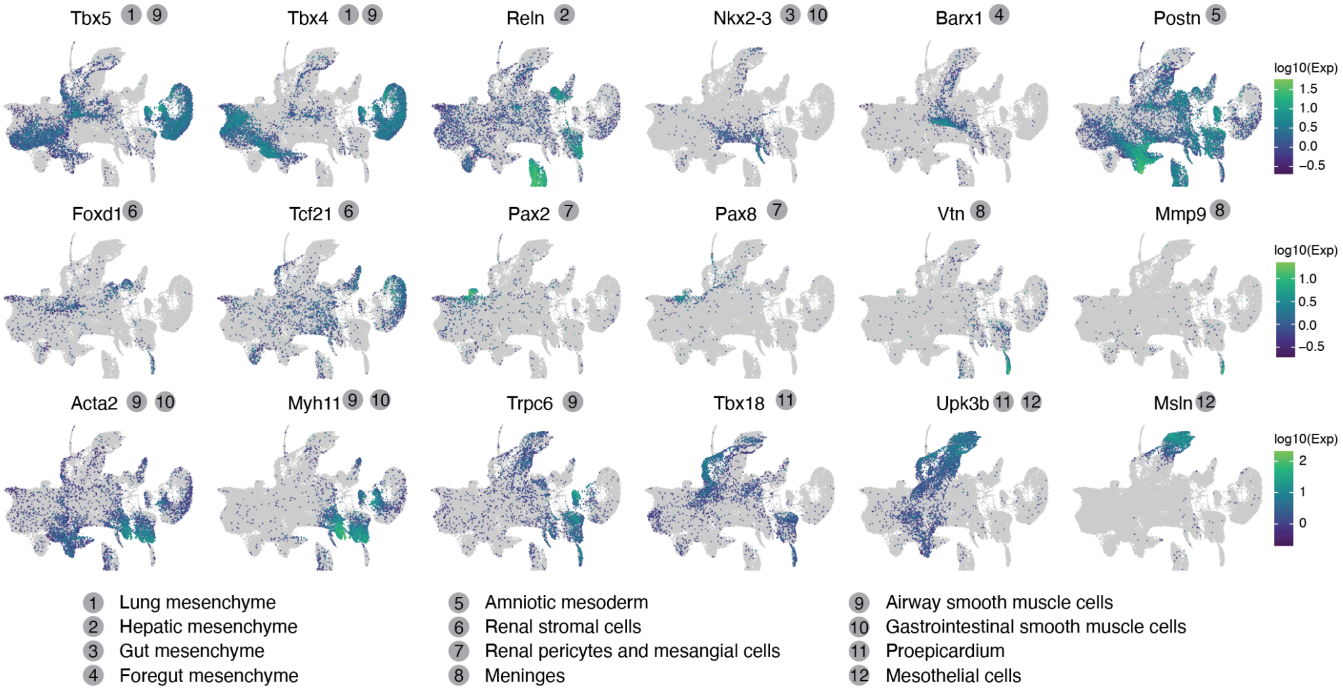

Supplementary Figure 8. Transcriptional heterogeneity in mesenchyme. The same UMAP as in **Fig. 3f**, colored by expression of marker genes which appear specific to lung mesenchyme (*Tbx5*+, *Tbx4*+), hepatic mesenchyme (*Reln*+), gut mesenchyme (*Nkx2-3*+), foregut mesenchyme (*Barx1*+), amniotic mesoderm (*Postn*+), renal stromal cells (*Foxd1*+, *Tcf21*+), renal pericytes and mesangial cells (*Pax2*+, *Pax8*+), meninges (*Vtn*+), airway smooth muscle cells (*Trpc6*+, *Tbx5*+), gastrointestinal smooth muscle cells (*Nkx2-3*+), proepicardium or mesothelium (*Msln*+). References for marker genes are provided in **Supplementary Table 12**.

bioRxiv preprint doi: https://doi.org/10.1101/2023.04.05.535726; this version posted April 5, 2023. The copyright holder for this preprint (which was not certified by peer review) is the author/funder, who has granted bioRxiv a license to display the preprint in perpetuity. It is made available under aCC-BY 4.0 International license.

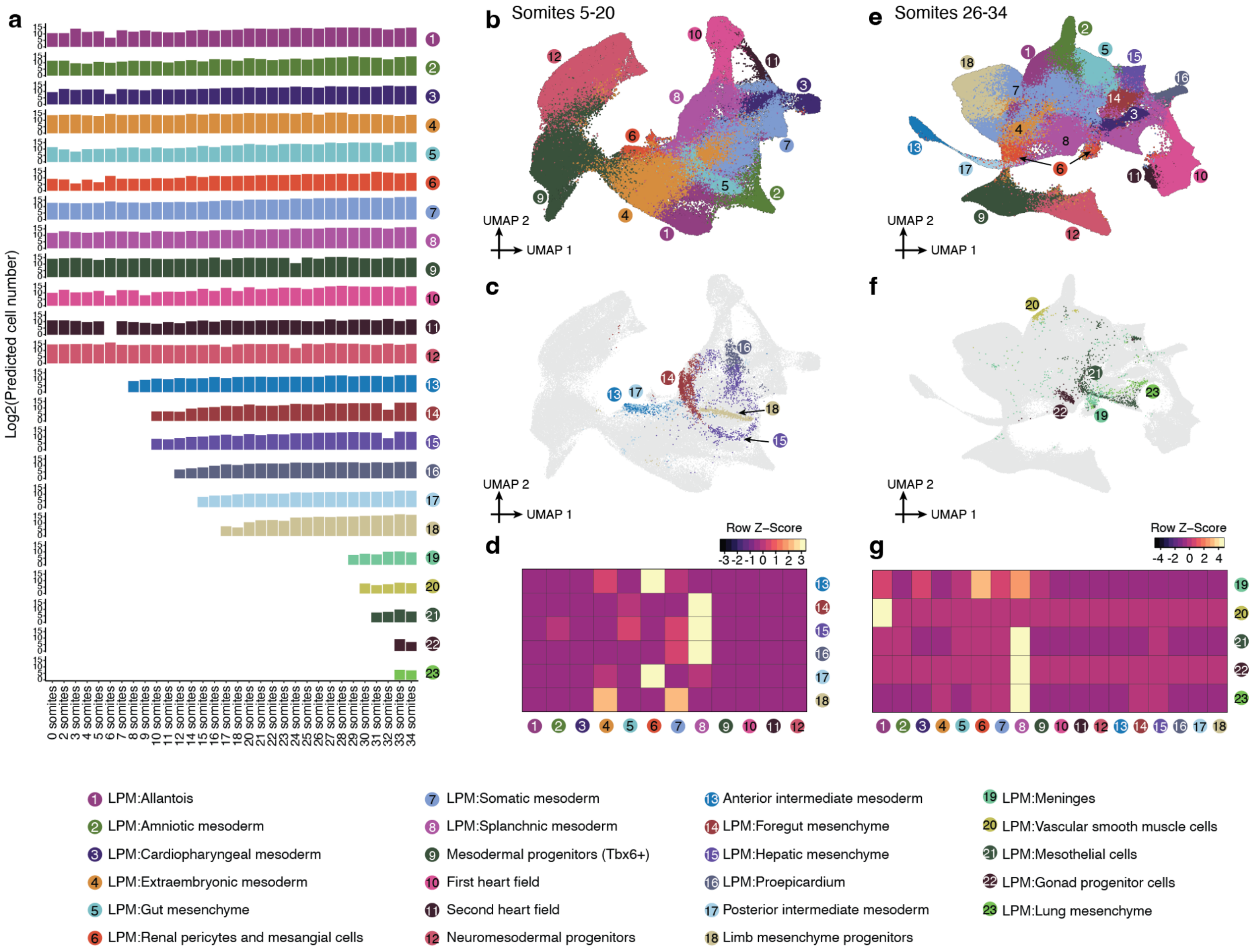

Supplementary Figure 9. The emergence of mesenchymal subtypes from the patterned mesoderm. a, The predicted absolute number (log2 scale) of cells of each mesoderm cell type at each somite count. The predicted absolute number was calculated by the product of its sampling fraction in the overall embryo and the predicted total number of cells in the whole embryo at the corresponding timepoint. Because cell numbers were only predicted for the broader bins (**Fig. 1e**), rather than individual somite counts, these were used for roughly corresponding sets (0-12 somite stage: E8.5; 14-15 somite stage: E8.75; 16-18 somite stage: E9.0; 20-23 somite stage: E9.25; 24-26 somite stage: E9.5; 27-31 somite stage: E9.75; 32-34 somite stage: E10.0). For each row, the first somite count with at least 10 cells assigned that cell type annotation is labeled, and all observations prior to that somite count are discarded. **b,** Re-embedded 2D UMAP of 110,753 cells from the selected cell types of mesoderm (clusters 1-12 as listed in panel a) from 5-20 somite stage embryos. **c,** The same UMAP as in panel b, but with inferred progenitor cells colored by derivative cell type with the highest mutual nearest neighbors (MNN) pairing score. **d,** Normalized MNN pairing score between mesodermal territories (column) and their inferred derivative cell types (row) from 5-20 somite stage embryos. The selected cell populations are first embedded into 30 dimensional PCA space, and then for individual derivative cell types, MNN pairs (k = 10 used for k-NN) between their earliest 500 cells (in absolute time) and cells from mesodermal territories are identified. **e,** Re-embedded 2D UMAP of 275,000 cells from the selected cell types of mesoderm (clusters 1-12 as listed in panel a) from 26-34 somite stage embryos. **f,** The same UMAP as in panel e, but with inferred progenitor cells colored by derivative cell type with the highest MNN pairing score. **g,** Normalized MNN pairing score between mesodermal territories (column) and their inferred derivative cell types (row) from 26-34 somite stage embryos. The selected cell populations are first embedded into 30 dimensional PCA space, and then for individual derivative cell types, MNN pairs (k = 10 used for k-NN) between their earliest 500 cells (in absolute time) and cells from mesodermal territories are identified.

bioRxiv preprint doi: https://doi.org/10.1101/2023.04.05.535726; this version posted April 5, 2023. The copyright holder for this preprint (which was not certified by peer review) is the author/funder, who has granted bioRxiv a license to display the preprint in perpetuity. It is made available under aCC-BY 4.0 International license.

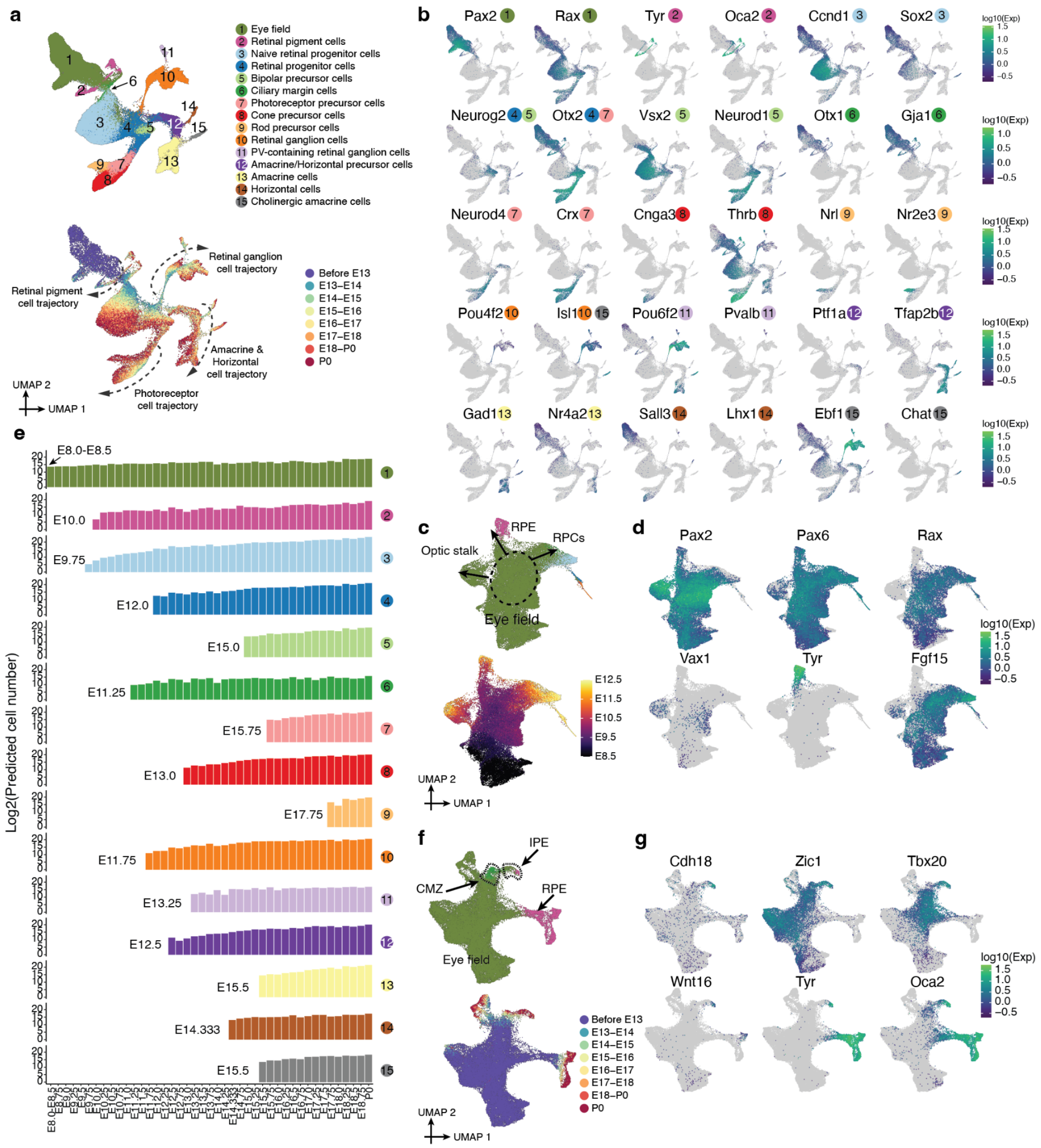

Supplementary Figure 10. The timing and trajectories of retinal development. a, Re-embedded 2D UMAP of 160,834 cells corresponding to the retinal development from E8 to P0. Cells are colored by either their initial annotations (top) or timepoint (bottom, after downsampling to a uniform number of cells per time window). Arrows highlight four of the main trajectories observed. Same as **Fig. 4a** except 2D instead of 3D projection. **b,** The same UMAP as in panel a, colored by gene expression of marker genes for each annotated retinal cell type. References for marker genes are provided in **Supplementary Table 5**. **c,** Re-embedded 2D UMAP of the subset of cells in panel a from stages <= E12.5. Cells are colored by either their initial annotations (top) or timepoint (bottom). **d,** The same UMAP as in panel c, colored by gene expression of markers of retinal progenitor cells RPCs (*Pax2*+, *Pax6*+, *Rax*+, *Fgf15*+)[69], RPE (*Tyr*+)[70], and the optic stalk (*Pax2*+, *Vax1*+, *Rax*-)[71]. **e,** The predicted absolute number (log2 scale) of cells of each retinal cell type at each timepoint. The predicted absolute number was calculated by the product of its sampling fraction in the overall embryo and the predicted total number of cells in the whole embryo at the corresponding timepoint (**Fig. 1e**). For each row, the first timepoint with at least 10 cells assigned that cell type annotation is labeled, and all observations prior to that timepoint are discarded. **f,** Re-embedded 2D UMAP of a subset of cells in panel a corresponding to eye field, RPE and CMZ. Cells are colored by either their initial annotations (top) or timepoint (bottom). **g,** The same UMAP as in panel f, colored by gene expression of marker genes for IPE[72] or pigment epithelium more generally (*Tyr* & *Oca2*). RPE: retinal pigment epithelium. CMZ: ciliary marginal zone. RPCs: retinal progenitor cells. IPE: iris pigment epithelium.

bioRxiv preprint doi: https://doi.org/10.1101/2023.04.05.535726; this version posted April 5, 2023. The copyright holder for this preprint (which was not certified by peer review) is the author/funder, who has granted bioRxiv a license to display the preprint in perpetuity. It is made available under aCC-BY 4.0 International license.

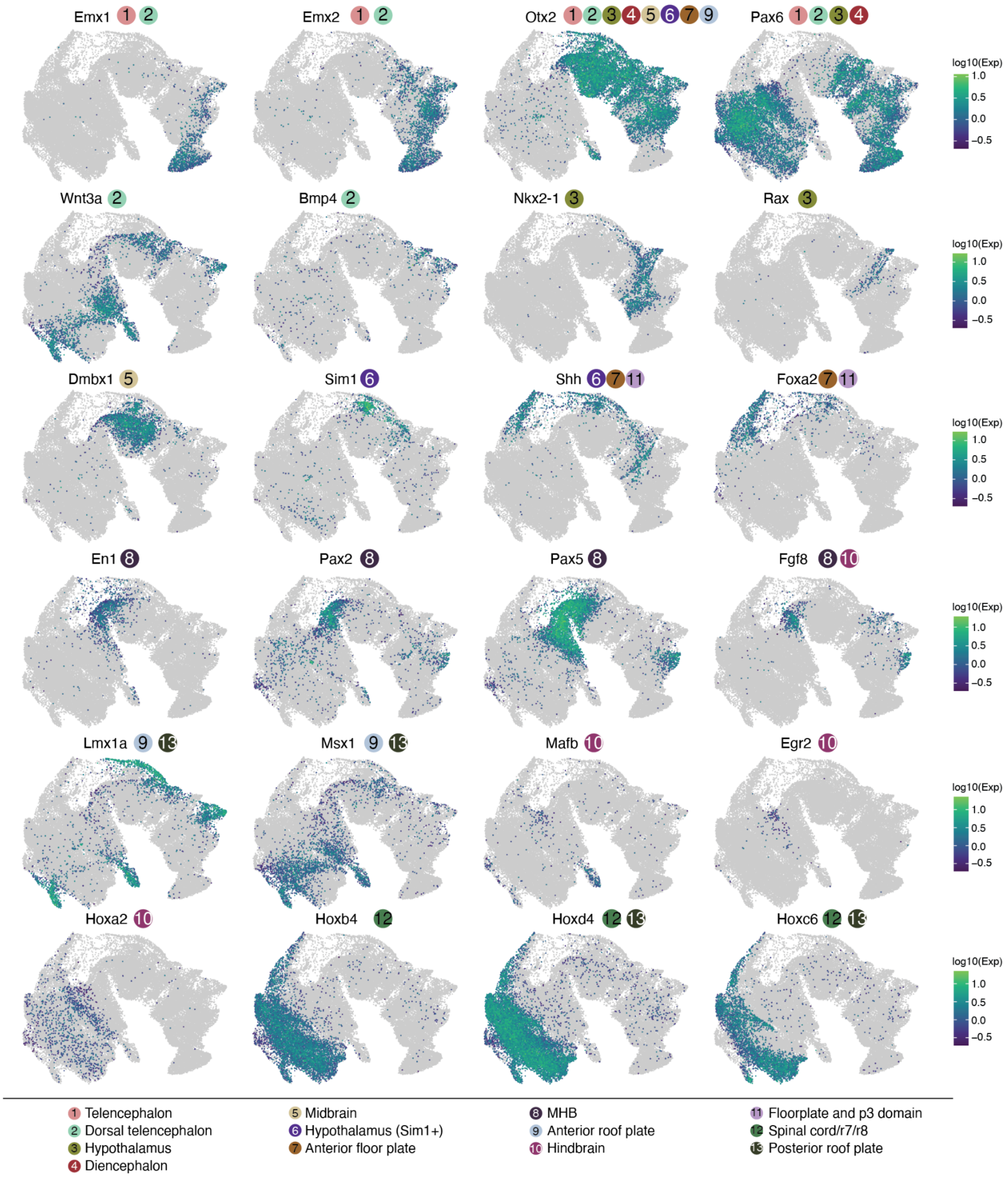

Supplementary Figure 11. Marker gene expression for different neuroectodermal territories. The same UMAP as in **Fig. 5a**, colored by gene expression of marker genes for each neuroectodermal territory. References for marker genes are provided in **Supplementary Table 5**.

bioRxiv preprint doi: https://doi.org/10.1101/2023.04.05.535726; this version posted April 5, 2023. The copyright holder for this preprint (which was not certified by peer review) is the author/funder, who has granted bioRxiv a license to display the preprint in perpetuity. It is made available under aCC-BY 4.0 International license.

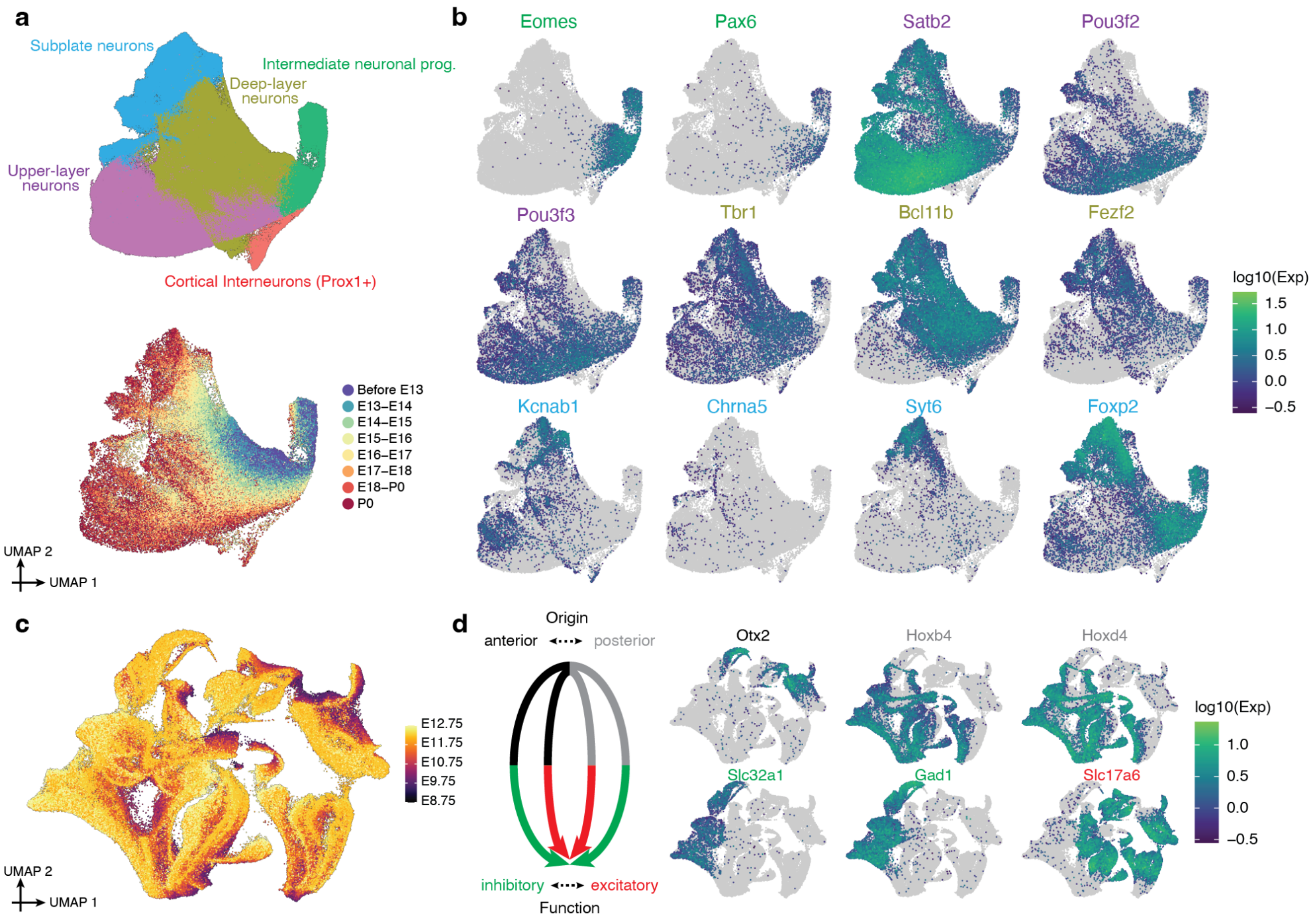

Supplementary Figure 12. Subtypes of intermediate neuronal progenitors, glutamatergic & GABAergic neurons. a, Re-embedded 2D UMAP of 628,251 cells within the intermediate neuronal progenitors major cell cluster, colored by either cell type (top) or developmental stage (bottom, after downsampling to a uniform number of cells per time window). **b,** The same UMAP as in panel a, colored by gene expression of marker genes which appear specific to intermediate neuronal progenitors (*Eomes*+, *Pax6*+), upper-layer neurons (*Satb2*+, *Pou3f2*+, *Pou3f3*+), deep-layer neurons (*Tbr1*+, *Bcl11b*+, *Fezf2*+), or subplate neurons (*Kcnab1*+, *Chrna5*+, *Syt6*+, *Foxp2*+). References for marker genes are provided in **Supplementary Table 5**. **c,** The same UMAP as in **Fig. 5e**, with cells colored by timepoints. **d,** Left: Neuronal subtypes shown in **Fig. 5e** originate from anterior vs. posterior of neuroectoderm, and then subsequently display inhibitory vs. excitatory functions after differentiation. Right: The same UMAP as in **Fig. 5e**, colored by gene expression of marker genes which appear specific to anterior (*Otx2*+)[79] vs. posterior (*Hoxb4*+, *Hoxd4*+)[80] origins, or inhibitory (*Slc32a1*+, *Gad1*+)[81] vs. excitatory (*Slc17a6*+)[82] functions.

bioRxiv preprint doi: https://doi.org/10.1101/2023.04.05.535726; this version posted April 5, 2023. The copyright holder for this preprint (which was not certified by peer review) is the author/funder, who has granted bioRxiv a license to display the preprint in perpetuity. It is made available under aCC-BY 4.0 International license.

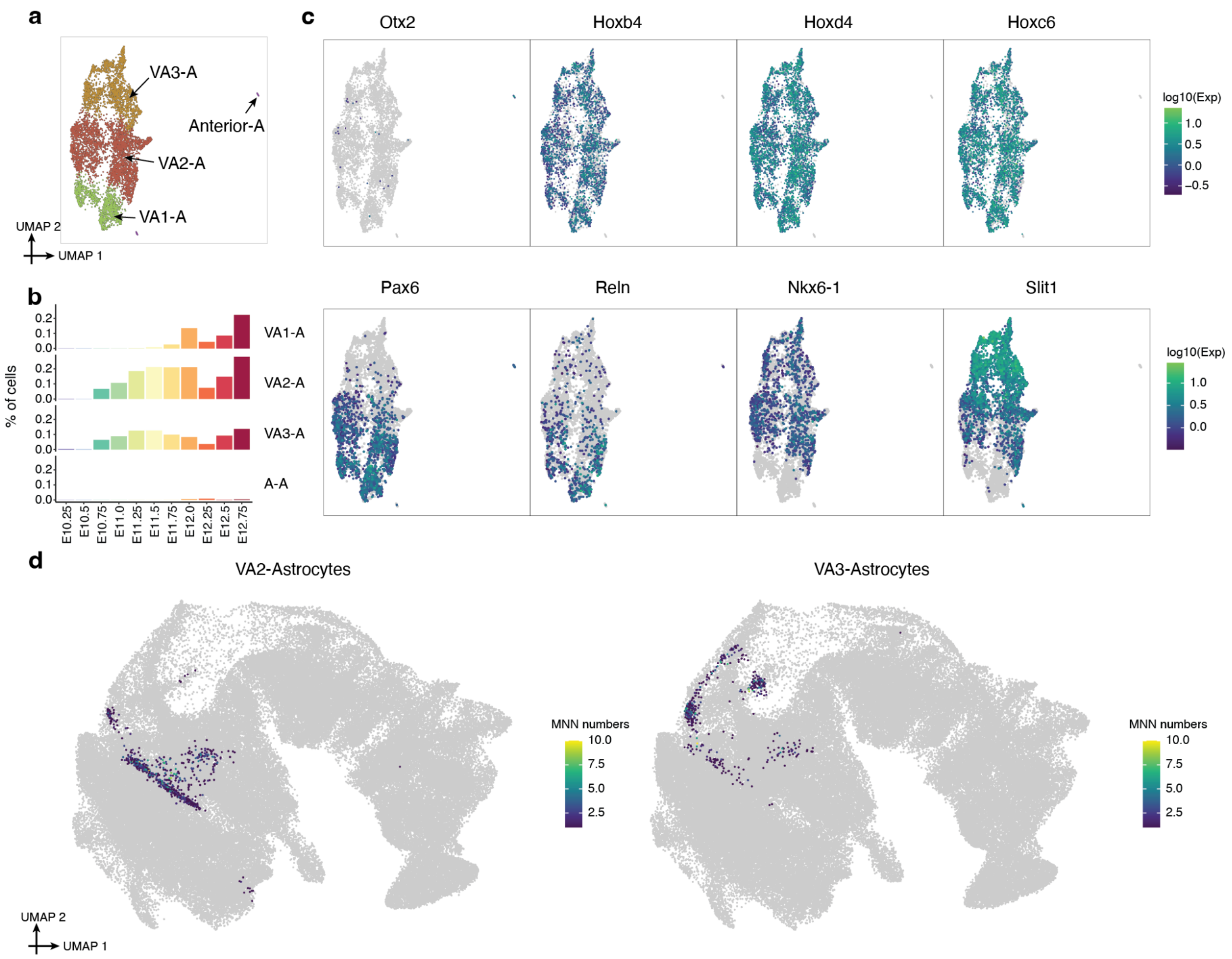

Supplementary Figure 13. Subtypes of early astrocytes and their inferred progenitors. a, Re-embedded 2D UMAP of 5,928 cells within the astrocytes from stages <E13. **b,** Composition of embryos from each 6-hr bin by different subpopulations of astrocytes. **c,** The same UMAP as in panel a, colored by gene expression of marker genes which appear specific to anterior (*Otx2*+) or posterior (*Hoxb4+, Hoxd4, Hoxc6*+) astrocytes, VA1-astrocytes (*Pax6*+, *Reln*+), VA2-astrocytes (*Pax6*+, *Reln*+, *Nkx6-1*+, *Slit1*+), and VA3-astrocytes (*Nkx6-1*+, *Slit1*+)[76]. **d,** The same UMAP of the patterned neuroectoderm as in **Fig. 5a**, with inferred progenitor cells of astrocytes colored by the frequency that has been identified as a MNN with either VA2-astrocytes (left) or VA3-astrocytes (right).

bioRxiv preprint doi: https://doi.org/10.1101/2023.04.05.535726; this version posted April 5, 2023. The copyright holder for this preprint (which was not certified by peer review) is the author/funder, who has granted bioRxiv a license to display the preprint in perpetuity. It is made available under aCC-BY 4.0 International license.

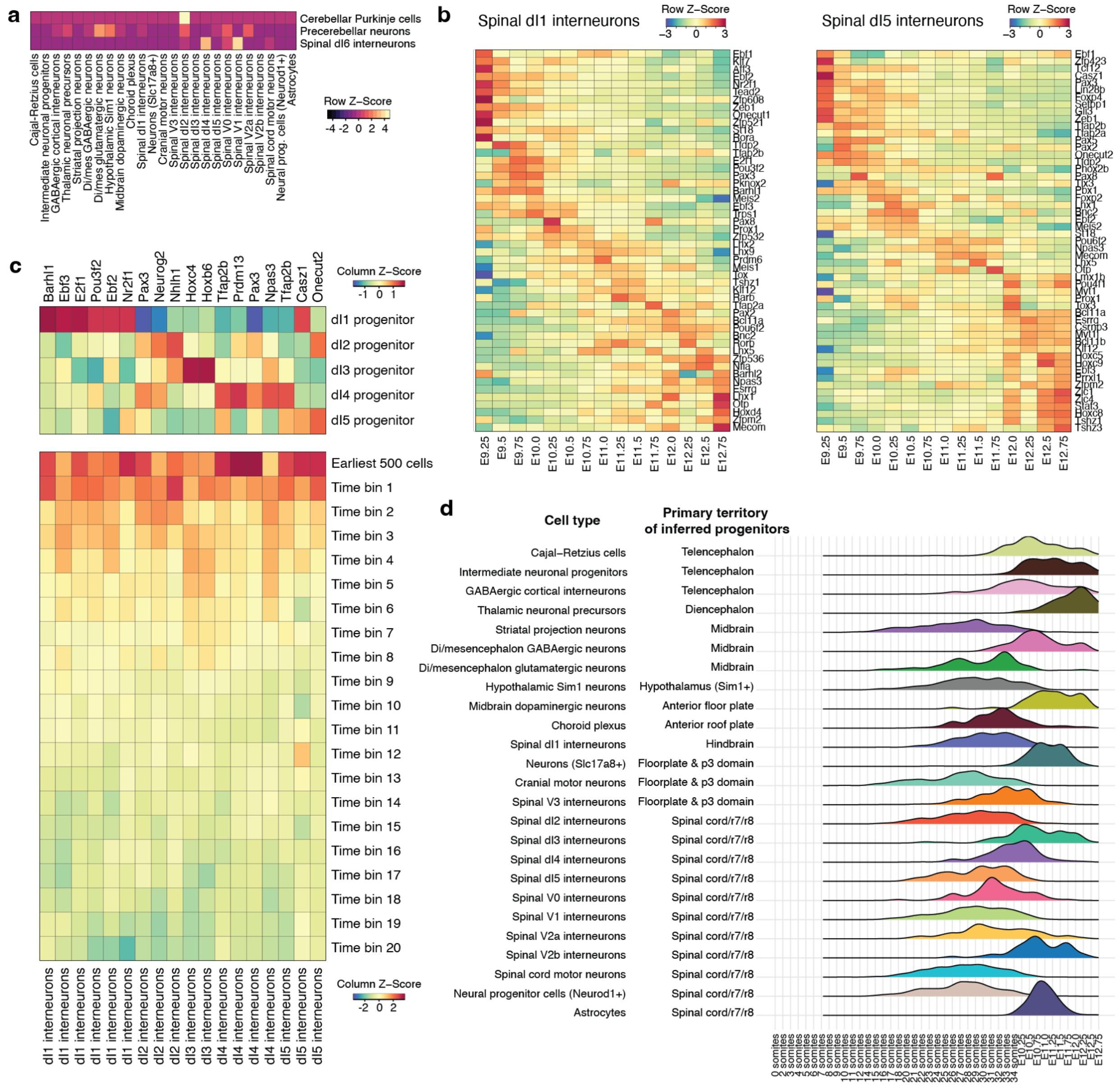

Supplementary Figure 14. The timing of neuronal subtype differentiation from the patterned neuroectoderm. a, For those three cell types (cerebellar Purkinje cells, precerebellar neurons, spinal dI6 interneurons) which were excluded in **Fig. 5d-e** due to having fewer than 50 MNN pairs, we performed a recursive mapping to identify whether they might share progenitors with another derived cell type, essentially repeating the analysis but attempting to map the earliest cells of these cell types to other derivative cell types rather than the patterned neuroectoderm. The heatmap shows the number of MNN pairs between pairwise cell types. In brief, this analysis suggests that spinal dI2 interneurons and cerebellar astrocytes share progenitors, while the progenitors of the other two re-analyzed cell types remain ambiguous. **b,** Gene expression across timepoints, for the specific TF markers of spinal dI1 (left) or spinal dI5 (right) interneurons. **c,** Top: gene expression for 18 selected TFs, across progenitor cells of dI1-5 from the neuroectodermal territories. Bottom, gene expression for 18 selected TFs across 21 time bins for dI1-5 spinal interneurons in which the TF has been nominated as marker TF. For individual spinal interneurons (each column), the first time bin involves the earliest 500 cells, then the left cells break into 20 bins ordered by their timepoints and with the same number of cells in each bin. Only cells from stages <E13 are included. **d,** For each neuronal subtype in **Fig. 5i-j**, we selected the annotation in the patterned neuroectoderm to which the most inferred progenitors had been assigned, and plotted the distribution of timepoints for that subset of inferred progenitors.

bioRxiv preprint doi: https://doi.org/10.1101/2023.04.05.535726; this version posted April 5, 2023. The copyright holder for this preprint (which was not certified by peer review) is the author/funder, who has granted bioRxiv a license to display the preprint in perpetuity. It is made available under aCC-BY 4.0 International license.

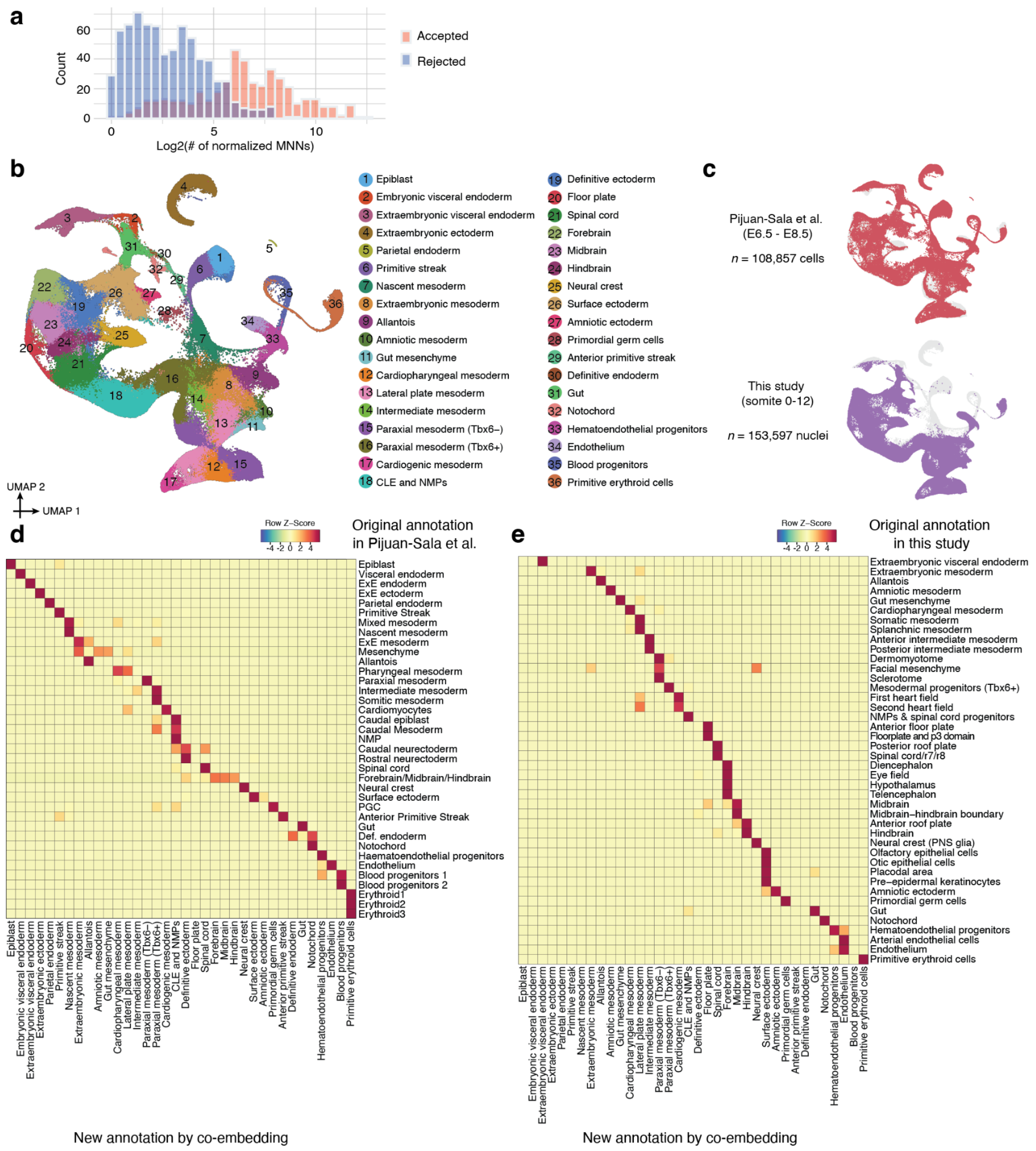

Supplementary Figure 15. Integration of scRNA-seq profiles from gastrulation and early somitogenesis to identify equivalent cell type nodes across datasets generated by distinct technologies. a, 1,155 edges with the number of normalized MNNs > 1 were manually reviewed for biological plausibility. Histogram of edges that were accepted or rejected as a function of normalized MNN score. **b,** 2D UMAP visualization of co-embedded cells, derived both from a gastrulation dataset based on cells from E6.5 to E8.5 generated on the 10x Genomics platform[6] (*n* = 108,857 cells) and the earliest ~1% of this dataset (0-12 somite stage embryos) generated by sci-RNA-seq3 (*n* = 153,597 nuclei), after batch correction[51]. This is essentially an updated version of an analysis that we have done previously[11]. We performed clustering and cell type annotation on the integrated co-embedding, as shown. **c,** The same UMAP as in panel b is shown twice, with colors highlighting cells/nuclei from Pijuan-Sala's dataset[6] (top) or early somitogenesis[11] (bottom). **d,** For cells from the original Pijuan-Sala's dataset[6], we quantify and display the overlap between the original annotations and the new annotations shown in panel b. For each row, the proportions of cells that are distributed across each column are transformed to z-score. **e**, For nuclei from the early somitogenesis embryos[11], we quantify and display the overlap between the original annotations and the new annotations shown in panel b. These mappings were the basis for dataset equivalence edges between the "gastrulation" and 12 "organogenesis & fetal development" subsystems. For each row, the proportions of cells that are distributed across each column are transformed to z-score. CLE: Caudal lateral epiblast. NMPs: Neuromesodermal progenitors.

bioRxiv preprint doi: https://doi.org/10.1101/2023.04.05.535726; this version posted April 5, 2023. The copyright holder for this preprint (which was not certified by peer review) is the author/funder, who has granted bioRxiv a license to display the preprint in perpetuity. It is made available under aCC-BY 4.0 International license.

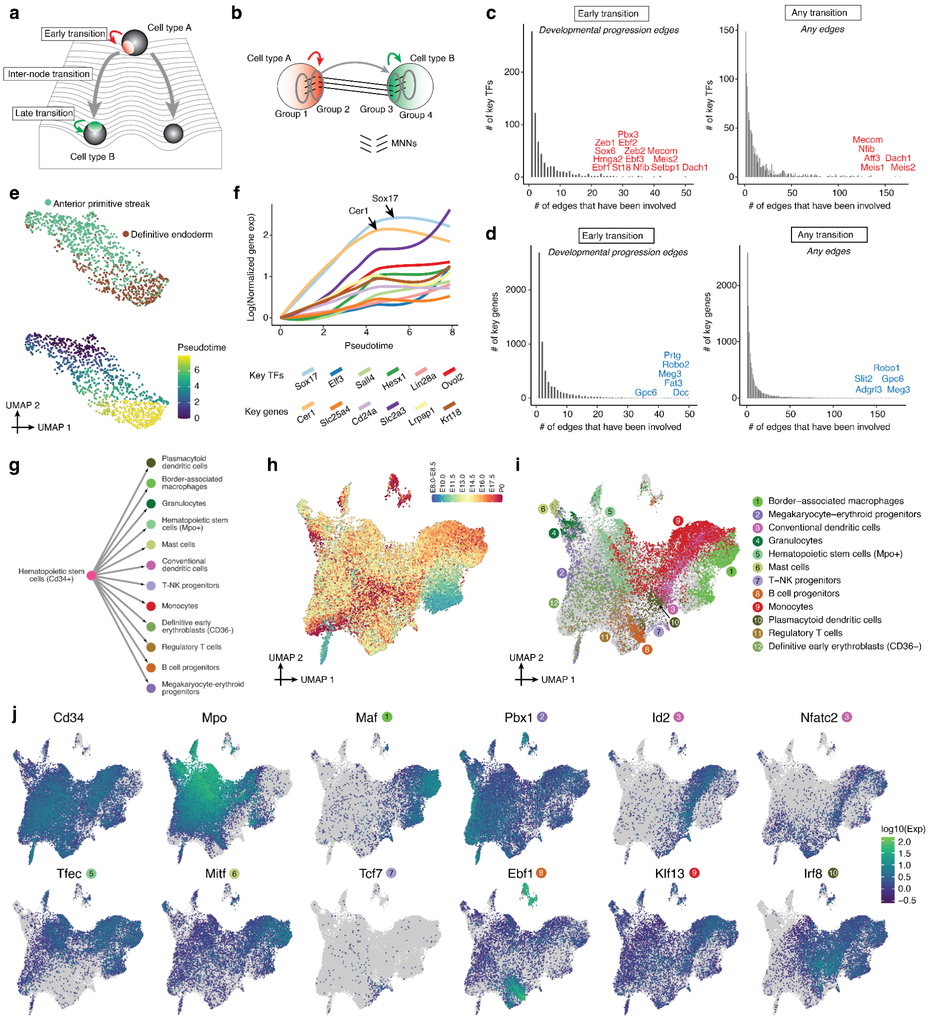

Supplementary Figure 16. Systematic nomination of TFs and other genes for cell type specification. a, A Waddington landscape cartoon illustrating how a cell type transition might be broken into three phases. **b,** Given a directional edge between two nodes, A→B, we identified the subset of cells within each node that were either MNNs of the other cell type (inter-node; groups 2 & 3) or MNNs of those cells (intra-node; groups 1 & 4). If A→B, this effectively models the transition as group 1→2→3→4. **c,** Histograms of the number of edges in which TFs are differentially expressed. The left histogram counts only genes when they are differentially expressed across the early phase of an developmental progression edge, while the right histogram counts genes when they are differentially expressed in any phase of all edges. **d**, Same as panel c, but for all genes rather than only TFs. **e,** Re-embedded 2D UMAP of 988 cells participating in groups 1-4 of the transition from anterior primitive streak → definitive endoderm. Cells are colored by either cell type annotations (top) or estimated pseudotime (bottom) using *Monocle*3[7]. **f,** For cells in panel e, normalized gene expression of selected genes are plotted as a function of estimated pseudotime. Gene expression values were calculated from original UMI counts normalized to total UMIs per cell, followed by natural-log transformation. The line of gene expression was plotted by the *geom_smooth* function in ggplot2. We manually added an offset based on their expression at pseudotime = 0 to the y-axis for individual genes. **g,** A sub-graph of **Fig. 6g**, including hematopoietic stem cells (Cd34+) and 12 cell type nodes which appear derived from it. **h,** Re-embedded 2D UMAP of 37,750 cells from hematopoietic stem cells (Cd34+), colored by developmental stage (after downsampling to a uniform number of cells per stage). **i,** The same UMAP as in panel h, but with inferred progenitor cells (the cells participating in the MNNs that support the edges) colored by derivative cell type with the most frequent MNN pairs. **j,** The same UMAP as in panel h, colored by gene expression of selected top key TFs which were upregulated during the "early transition" for each derivative.

bioRxiv preprint doi: https://doi.org/10.1101/2023.04.05.535726; this version posted April 5, 2023. The copyright holder for this preprint (which was not certified by peer review) is the author/funder, who has granted bioRxiv a license to display the preprint in perpetuity. It is made available under aCC-BY 4.0 International license.

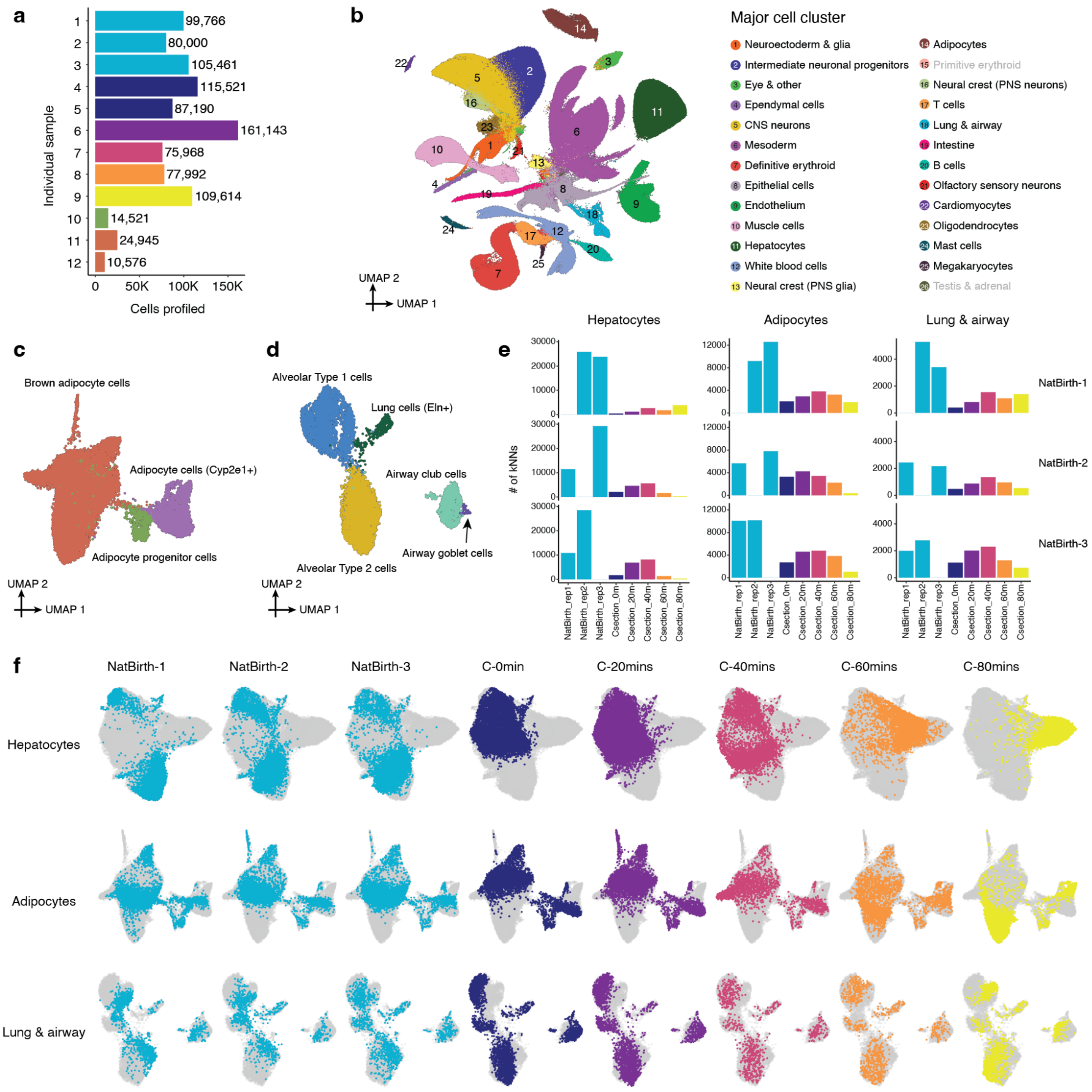

Supplementary Figure 17. Rapid shifts in transcriptional state occur in a restricted subset of cell types upon birth, and differ between vaginally and C-section delivered pups. a, The number of nuclei profiled for each animal shown in **Fig. 7c**. A small number of nuclei from additional fetal samples from the original set of experiments were also profiled for quality control (samples 10-12). **b,** 2D UMAP visualization of the birth-series dataset (*n* = 962,697 cells). Colors correspond to 26 major cell cluster annotations (**Fig. 1e**). Two major cell clusters (the primitive erythroid and testis & adrenal major cell clusters) shown in the original dataset but missed here are highlighted in gray. Primitive erythroid cells are not present at these timepoints and testis & adrenal cells are collapsed to the epithelial cells major cell cluster due to their low numbers. **c,** Re-embedded 2D UMAP of 19,696 cells of the adipocyte major cell cluster. **d,** Re-embedded 2D UMAP of 7,986 cells of the lung & airway major cell cluster. **e,** For these three major cell clusters, we co-embedded cells from three vaginally delivered pups (samples 1-3 in **Fig. 7c**) and six pups delivered by C-section (samples 4-9 in **Fig. 7c**), followed by subsetting a uniform number of cells per sample. For cells from each of the three vaginally delivered pups, we calculated the number of their 10 nearest neighbors in the PCA embedding (*n* = 30 dimensions) from other samples. **f,** Re-embedded 2D UMAP of cells from these three major cell clusters, based on cells from three vaginally delivered pups and six pups delivered by C-section. For each row, the same UMAP is shown multiple times, with colors highlighting cells from individual pups (or two pups, in the case of the 0-min C-section timepoint).

bioRxiv preprint doi: https://doi.org/10.1101/2023.04.05.535726; this version posted April 5, 2023. The copyright holder for this preprint (which was not certified by peer review) is the author/funder, who has granted bioRxiv a license to display the preprint in perpetuity. It is made available under aCC-BY 4.0 International license.

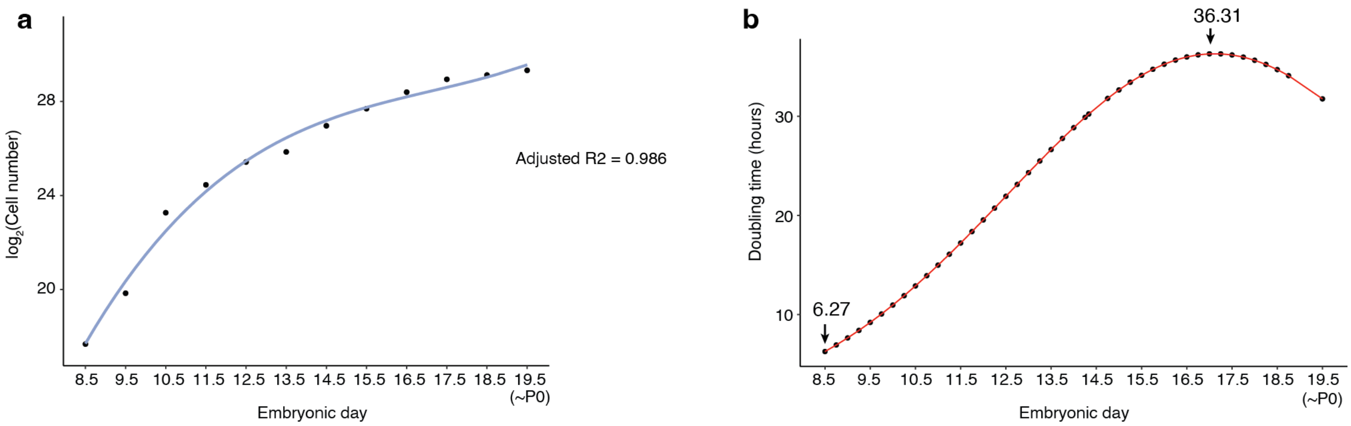

Supplementary Figure 18. Quantitatively estimating cell number for individual mouse embryos as a function of developmental stage. a, Based on the experimentally estimated cell numbers of the 12 embryos (ranging from E8.5 to P0), we applied polynomial regression (degree = 3) to fix a curve across embryos between the embryonic day and log2-scaled cell number. P0 was treated as E19.5 in the model. **b,** The estimated “doubling time” of the total cell number in a whole mouse embryo are plotted as a function of timepoints. The timepoints with the longest (E17.0) and shortest (E8.5) estimated “doubling times” are highlighted.
